# A comparison of three techniques (local anesthetic deposited circumferential to vs. above vs. below the nerve) for ultrasound guided femoral nerve block

**DOI:** 10.1186/1471-2253-14-6

**Published:** 2014-01-25

**Authors:** Szilárd Szűcs, Didier Morau, Syed F Sultan, Gabriella Iohom, George Shorten

**Affiliations:** 1 Department of Anaesthesia, Intensive Care and Pain Medicine, Cork University Hospital and University College Cork, Cork, Ireland; 2 Department of Anaesthesia and Traumatology, Centre Hospitalier, Universitaire de Montpellier, DARA, France

**Keywords:** Optimal positioning of the local anesthetic, Femoral nerve block

## Abstract

**Background:**

Fractured neck of femur generally requires operative fixation and is a common cause of admission to hospital. The combination of femoral nerve block and spinal anesthesia is a common anesthetic technique used to facilitate the surgical procedure. The optimal disposition of local anesthetic (LA) relative the femoral nerve (FN) has not been defined. Our hypothesis was: that the deposition of LA relative to the FN influences the quality of analgesia for positioning of the patient for performance of spinal anesthesia. The primary outcome was verbal rating (VRS) pain scores 0–10 assessed immediately after positioning the patient to perform spinal anesthesia.

**Methods:**

With Institutional ethical approval and having obtained written informed consent from each, 52 patients were studied. The study was registered with ClinicalTrials.gov (NCT01527812). Patients were randomly allocated to undergo to one of three groups namely: intention to deposit lidocaine 2% (15 ml) i. above (Group A), ii. below (Group B), iii. circumferential (Group C) to the FN. A blinded observer assessed i. the sensory nerve block (cold) in the areas of the terminal branches of the FN and ii. VRS pain scores on passive movement from block completion at 5 minutes intervals for 30 minutes. Immediately after positioning the patient for spinal anesthesia, VRS pain scores were recorded.

**Results:**

Pain VRS scores during positioning were similar in the three groups [Above group/Below group/Circumferential group: 2(0–9)/0(0–10)/3(0–10), median(range), p:0.32]. The block was deemed to have failed in 20%, 47% and 12% in the Above group, Below group and Circumferential group respectively. The median number of needle passes was greater in the Circumferential group compared with the Above group (p:0.009). Patient satisfaction was greatest in the Circumferential group [mean satisfaction scores were 83.5(19.8)/88.1(20.5)/93.8(12.3), [mean(SD), p=0.04] in the Above, Below and Circumferential groups respectively.

**Conclusions:**

We conclude that there is no clinical advantage to attempting to deposit LA circumferential to the femoral nerve (relative to depositing LA either above or below the nerve), during femoral nerve block in this setting.

## Background

Fractured neck of femur (FNF) often requires operative fixation and is a common cause of hospital admission for elderly patients. Spinal anesthesia is a technique which is commonly used for these cases and which is performed with the patient in the lateral decubitus position. Positioning the patient for spinal anesthesia can be very painful; avoidance of this discomfort is a common and unsolved problem for anesthetists.

Regional anesthesia is effective in alleviating pain due to trauma; it offers the advantage of producing localized and very effective pain relief [1]. Prior to positioning a patient with FNF for spinal anesthesia, femoral nerve blockade (FNB) can provide excellent pain relief and is generally well tolerated [2-5]. Ultrasound-guidance for peripheral nerve blockade is intended to improve the block success rate and it is increasingly used around. Casati and al. demonstrated a 42% decrease of effective dose (ED50%) by using ultrasound to localize the femoral nerve prior to FNB [6]. A recent editorial by Sites pointed out that the optimal disposition of the local anesthetic in ultrasound-guided peripheral nerve blockade has yet to be defined [7]. We currently employ different approaches in relation to injection of local anesthetic (LA) solution close to the femoral nerve. Firstly, one may attempt to position the LA circumferentially around the nerve. This technique requires several needle passes, which may cause patient additional, perhaps unnecessary discomfort. Another option is to inject the LA either above or below the nerve without changing the position of the tip of the needle, thereby minimizing the number of needle passes and, probably, the degree of patient discomfort. It is not known if this later approach (single injection above or below the nerve) results in an equivalent quality of sensory block and subsequent analgesia. The femoral nerve has separated into branches at this level and we assume that the spread of LA may influence the quality and the extent (distribution) of the block.

Our objective was to compare i. the analgesic efficacy of ultrasound-guided FNB to facilitate positioning of patients for spinal anesthesia and ii. block success when LA was positioned i. above ii. below or iii. circumferential to the femoral nerve.

## Methods

With the approval of the Clinical Research Ethics Committee of the Cork Teaching Hospitals (ECM 4 (zz) 08/12/09.) and having registered the trial at ClinicalTrials.gov (NCT01527812), a prospective, double blinded, randomized study of patients undergoing operative fixation of FNF at the Cork University Hospital was undertaken between December 2009 and November 2011. The patients were randomly allocated using a random number sequence and sealed envelopes. Written, informed consent was obtained from each patient.

Patients with FNF, American Society of Anesthesiologists grades I to III and aged >50 years, were invited to participate in the study. Exclusion criteria were patient refusal, the presence of more than one fracture; Mini-Mental Score <22 (Additional file 1); coagulation disorders; head injury; history of loss of consciousness; acute heart failure; allergy to lidocaine; skin lesions/infection at block site; and renal dysfunction. Patients with evidence of systemic infections (clinically defined or elevated C-reactive protein levels, leucocytosis, or body temperature higher than 37.8°C) were also excluded.

In all patients, an experienced anesthetist performed the ultrasound guided FNB. A 5 cm, 6–13 MHz linear transducer probe (Sonosite Turbo M, Bothwell WA, USA) was used to locate the nerve. For optimal visualization of the femoral nerve the transducer was applied transversely to the thigh below the inguinal crest. After examination of the anatomy of the femoral artery, the femoral nerve was identified at a level immediately above the deep femoral artery branch bifurcation. A 22 G 50 mm Stimuplex BBraun needle was used. After identification of the nerve and fascia around the nerve, the skin was infiltrated with local anesthetic ( 0.2 ml lidocaine 1%) on the lateral aspect of the thigh, 1 cm lateral to the lateral edge of the transducer. The needle was inserted in-plane from lateral to medial and advanced toward the lateral aspect of the femoral nerve.

**Figure 1:**
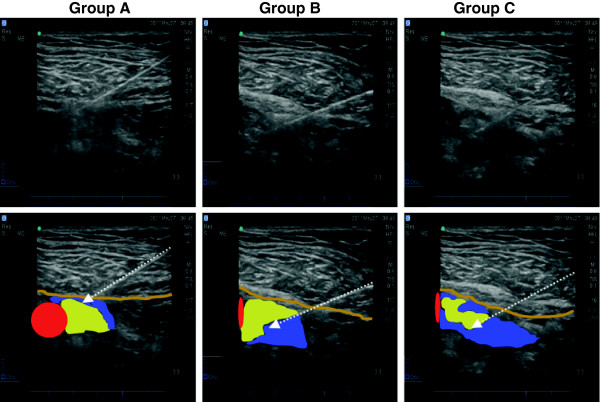
**Composite figure of the femoral nerve block.** Representative images depicting the anatomy: fascia iliaca (light brown lines), femoral artery (red), femoral nerve (yellow), needle (arrow) and local anesthetic (blue) position in Group **A, B** and **C**.

For all patients, lidocaine 2% 15 ml was administered to perform ultrasound guided FNB. We used lidocaine, because it had a short onset time and our aim it was to facilitate positioning for performing spinal anesthesia in the shortest time. For patients allocated to the “Above” group (*Group A)* the LA was injected below (i.e. deep to) the fascia iliaca and above (i.e. superficial to) the femoral nerve; for patients allocated to the “below” group (*Group B)*, the LA was injected below the femoral nerve and above the fascia of the iliopsoas muscle and for those patients allocated to the circumferential group (*Group C),* circumferential spread was achieved with multiple injections around the nerve (Figure 1).

An independent blinded observer (not present during performance of the block) assessed the extent and degree of sensory blockade using a modified Bromage score (cold, mildly cold and just spray) at 5 minute intervals during the initial 30 minutes after block completion. Sensory perception was assessed using cold (ethyl-chloride spray) spray on the skin in the lateral, frontal, medial side of the thigh and medial side of the leg corresponding to common distributions of the terminal branches of the femoral nerve.

Our primary outcome parameter was pain, evaluated using verbal rating (VRS) pain score (0–10) immediately after positioning the patient (lateral decubitus with operative side superior/independent) for spinal anesthesia.

We recorded each patient’s pain (also using a VRS pain score 0–10) on passive movement of the fractured limb (elevating up to 30 degrees from the supine position or to patient tolerance from the resting position). When the patient reported VRS < 4 during the passive movement of the limb, the sensory block was deemed adequate and the patient was positioned for spinal anesthesia. In the event that cold perception was still present, assessment was continued up to 30 minutes after block completion (if the spinal is not injected until this time). Block failure was defined as failure to achieve a VRS score of < 4 within 30 minutes of FNB completion. In these cases, additional opioid medication and/or sedation were administered in order to optimize positioning for spinal anesthesia and these patients were excluded from further data collection.

We recorded the times at which the patients arrived in the anesthetic induction room, ultrasound-guided FNB started (i.e. skin infiltration with LA) and completion of patient positioning for spinal anesthesia.

Spinal anesthesia was performed using standard aseptic technique; isobaric bupivacaine 0.5% were administered at a dose indicated by the responsible clinician. Patient satisfaction was assessed using a 100 mm linear visual analogue scale (VAS) during the surgical procedure and immediately after arriving to the recovery area. Patients were also asked in the recovery area if, given the option, they would choose the same analgesic modality again.

Untoward or adverse events were recorded by the responsible clinician (anesthetist) on a dedicated data sheet.

## Statistical analysis

Sample size calculation was limited by the absence of historical data on the degree of pain patients with FNF experienced while being positioned for spinal anesthesia. It was arbitrarily decided to proceed on the basis that at least 20 patients/group would be required to demonstrate a clinically relevant effect size. Collected data were examined for normality. Normally distributed variables were tested between groups using ANOVA and t-test, non-normal data were analyzed using the non-parametric Kruskal-Wallis and Mann–Whitney U test. Categorical variables were tested using Chi-squared tests. P < 0.05 was considered significant.

## Results

Sixty patients were recruited to this study of whom 52 were managed per protocol. Seven patients were excluded because of breaches of study protocol. For instance, one patient (Group A) developed fast atrial fibrillation after performing spinal anesthesia resulting in hemodynamic instability and cancellation of surgery. In one case (Group C), the anesthetist who performed the ultrasound-guided FNB had difficulties in visualizing the femoral nerve, and a nerve stimulator was used to confirm its position.

**Figure 2:**
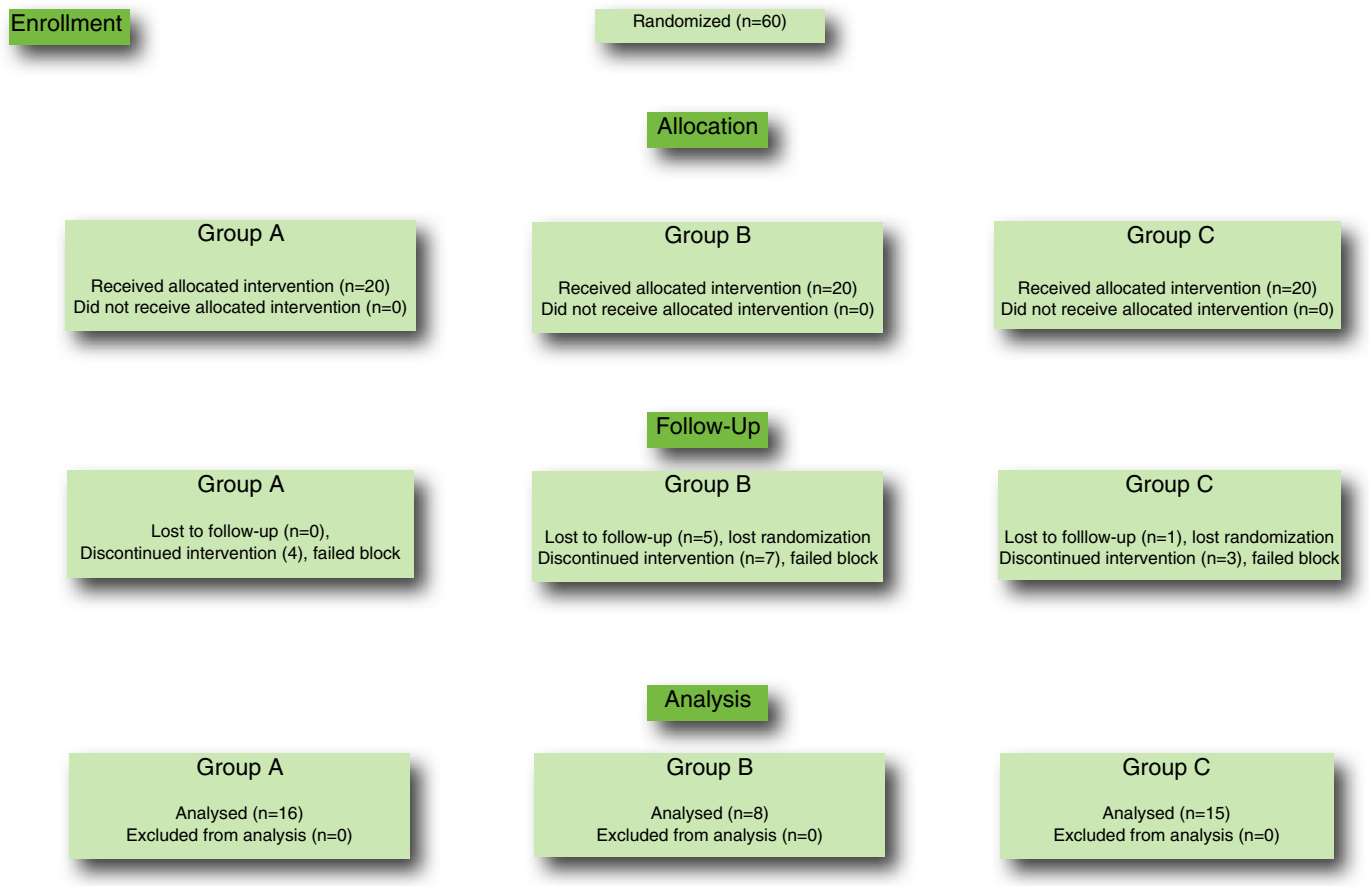
Enrollment.

Patient characteristics were similar in the three groups (Table 1). Block failure (as defined above) occurred in four patients of 20 from Group A (20%) seven of 15 patients from Group B (46.7%) and three of 17 patients from Group C (17.6%) (Figure 2). The patients in whom the FNB block was deemed to have failed received iv. fentanyl, midazolam or propofol, at the discretion of the anesthetist responsible for their clinical care and excluded from further data collection.

**Table 1:** Patients demographic characteristics

	**Group A**	**Group B**	**Group C**	**(p-value)**
Gender (female/male)	11/5	4/4	7/8	(0.346)
Age (years, mean)	80.0	73.9	81.3	(0.343)
ASA status I/II/III	1/11/4	1/6/1	2/8/5	(0.666)
Procedure	10/6	6/2	10/5	(0.464)
(DHS, IMHS/hemiarhtroplasty)				
BMI (kg/m2) mean	23.16	25.29	25.51	(0.181)
Right/left	9/7	2/6	7/8	(0.212)

*ASA,* American Society of Anesthesiologist physical status; *DHS* Dynamic hip screw; *IMHS* Intramedullary hip screw.

**Figure 3:**
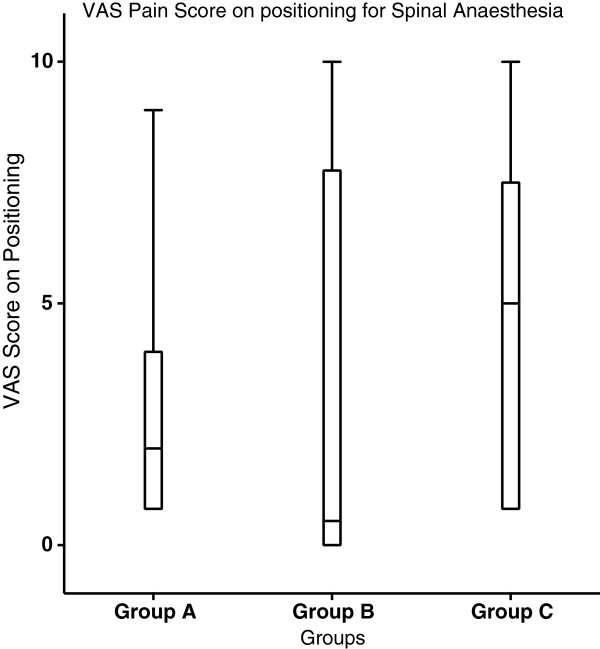
**VAS pain scores on positioning for spinal anesthesia.** VRS median pain scores at positioning to perform spinal anesthesia in Group A /Group B /Group C: 2.5(0–9)/3.2(0–10)/4.7(0–10), median(range) Kruskal-Wallis test p: 0.43) The box-and-whiskers plots show maximum and minimum values and 90th percentiles, lower and upper quartiles and the median (horizontal bar) for each group.

Pain scores on positioning for spinal anesthesia were similar in the three groups [VRS pain scores in the Group A/Group B/Group C: 2(0–9)/0(0–10)/3(0–10), median (range), Kruskal-Wallis test p:0.32)] (Figure 3). Patient satisfaction (VAS scores on arrival to the recovery room) was greater in Group C patients compared with those in Group A [Group C vs. Group A: 93.8(12.3) vs. 83.5(19.8), mean (SD), p: 0.01)]. The distribution of sensory block achieved was similar in the three groups.

Procedural pain (VRS), block procedural time, block onset time, the time to position for spinal anesthesia and spinal block performance time were also similar in the three groups (Table 2), during the FNB.

**Table 2:** Secondary outcomes (medians)

	**Group A**	**Group B**	**Group C**	**(p-value)**
Time till starting the USFNB after arrival to the induction room (min)	9.4	7.4	7.0	(0.886)
UGFNB procedure time (min)	3.3	3.4	4.6	(0.497)
Pain during UGFNB (VRS 0-10)	2.3	1.4	2.6	(0.64)
UGFNB onset time (min)	9.3	11.4	12.3	(0.49)
Turning time for spinal anesthesia after arrival in induction room (min)	32.1	29.1	35.0	(0.49)
Spinal performing time after arrival in induction room (min)	43.8	39.3	46.1	(0.62)
Sedation during spinal anesthesia, number of the patients (%)	2(12.5)	2(25)	3(20)	(0.73)

*VAS* Visual analogue scale; *VRS* Verbal rating score.

On one occasion, spinal anesthesia was converted to general anesthesia because the insertion of the spinal needle was impossible during multiple attempts. Fentanyl iv. and midazolam iv. were administered as clinically indicated during performance of spinal anesthesia (again at the discretion of the responsible anesthetist). In Group A, one patient received 20 microgram fentanyl iv. and two patients received 2 and 5 mg midazolam iv. In Group B, two patients received 20 and 25 microgram fentanyl iv. and one patient 1 mg midazolam iv. In Group C. one patient received fentanyl 20 mcg and two received 2 mg midazolam iv.

## Discussion

**Figure 4:**
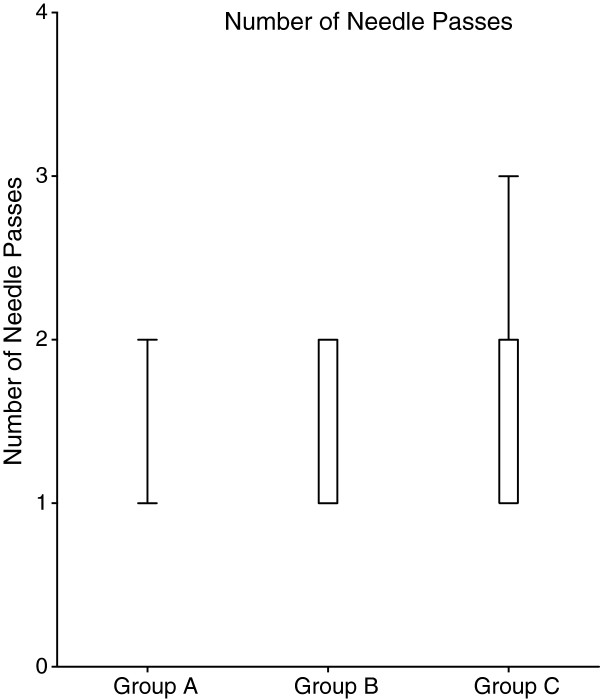
**Number of needle passes.** Boxplot showing distributions within each group of number of needle passes during ultrasound guided FNB. The median number of needle passes was statistically significantly higher in the Group C compared with the Group A (2.0 vs. 1.0, Mann–Whitney U between Groups C vs. A, p = 0.009). The Group B median was also higher than the group A median i.e. 1.5 but this was not significant.

The most important finding of this study is that the attempt to deposit LA circumferentially around the femoral nerve offered no clinical advantage (in terms of pain on positioning for spinal anesthesia) relative to attempting to deposit LA only above (i.e. superficial to) the nerve. The latter approach resulted in fewer needle passes during performance of the block (Figure 4) and was associated with greater patient satisfaction on arrival to the postoperative recovery room.

We believe that our understanding of the determinants of spread of LA administered during peripheral nerve blockade is grossly deficient. The evidence and our understanding of the equivalent determinants when LA is administered for neruraxial block is greater but still incomplete. Our study attempts to apply scientific rigor to a clinical (i.e. applied) question without making unsupported assumptions.

Previous studies have demonstrated that ultrasound is a reliable method of detecting injectate spread in a gelatin phantom model [8]. It has also been shown that ultrasound-guided circumferential injection of local anesthetic around the sciatic nerve can improve the rate of sensory block [9]. It has been demonstrated that fascia iliaca block is more efficacious than i.v. alfentanil in terms of facilitating the lateral position for spinal anesthesia [10]. FNB has been shown to be superior (compared with i.v. administration of fentanyl) in facilitating the sitting position for spinal anesthesia in patients undergoing surgery for femoral shaft fractures [11]. A recent investigation of the influence of catheter tip positioning during continuous FNB in healthy volunteers concluded that anterior (vs. posterior) placement increased cutaneous sensory block, without a concurrent relative increase in motor block [12]. Ours was a clinical investigation aimed at providing useful practical information to clinicians seeking to optimize conditions for positioning of patients prior to FNF surgery. Thus, in addition to the cutaneous sensory effects, we considered that articular pain may contribute to the discomfort experienced by these patients. The posterior division of the femoral nerve gives articular branches to the hip and knee [13]. Therefore we believed that it was possible that deposition of LA inferior, just below the femoral nerve at the level described could effect greater sensory block via these articular branches. Kullenberg et al. reported that FNB could have other beneficial outcomes in this patient group, including earlier times to postoperative mobilization and less cognitive impairment [14]. Ultrasound-guided FNB is feasible to perform in the emergency department and significant and sustained decreases in pain scores were achieved with this technique [15].

The relatively great incidence of block failure we report may be a function of the strict definition of failure we applied. There is well documented variation in sensory innervation of the hip joint (with differing contribution across individuals from femoral, sciatic and obturator nerves) [13]. The relatively small sample size may also have contributed to this unexpected finding.

Our study has certain limitations. The data set wasn’t complete in every case. Certain patients received sedation after spinal anesthesia had been performed. Certain co-morbid factors may have influenced pain perception during positioning for spinal in these cases. For example, chronic obstructive pulmonary disease result in a longer duration of positioning the patient (and presumably greater discomfort). A negative finding of a clinical trial in which the sample size was relatively small and arbitrarily selected may be due to a Type II error.

## Conclusions

We believe that that is the first study which examines the association between distribution of injectate (or technique to achieve such a distribution) following FNB and defined clinical effect. We conclude that, in the clinical setting described, attempting to deposit LA circumferential to the femoral nerve (versus depositing it above/superficial to the nerve) confers no clinical advantage, results in a greater number of needle passes and therefore is not justified.

## Abbreviations

FNF: Fractured neck of femur; FN: Femoral nerve; FNB: Femoral nerve block; LA: Local anesthetic; VRS: Verbal rating score; VAS: Visual analog scale; ANOVA: Analysis of variance; SD: Standard deviation.

## Competing interests

The authors declare that they have no competing interests.

## Authors’ contributions

SS participated in the design of the study, performed statistical analysis, carried out data collection and led preparation of the manuscript. DM participated in the design of the study, performed randomization, carried out data collection and contributed to preparation of the manuscript. SFS participated in the design of the study, carried out data collection, GI participated in the design of the study, co-ordinated data collection and contributed to the preparation of the manuscript GS conceived of the research question addressed, co-ordinated the conduct of the study and contributed to the preparation of the manuscript. All authors read and approved the final manuscript.

## Pre-publication history

The pre-publication history for this paper can be accessed here:

http://www.biomedcentral.com/1471-2253/14/6/prepub

## Supplementary Material

Additional file 1 Mini Mental examination. Click here for file
