# Supplementary material for: A comparison of three techniques (local anesthetic deposited circumferential to vs. above vs. below the nerve) for ultrasound guided femoral nerve block
Source: BMC Anesthesiol. 2014 Jan 25;14:6. doi: 10.1186/1471-2253-14-6 (PMC3933203; doi:10.1186/1471-2253-14-6)
Supplement: Additional file 1 — Mini Mental examination. [file 1471-2253-14-6-S1.pdf]

Appendix 1.

Mini Mental Examination

Score	Section	Task
	Orientation	
5		**What is- year, season, date, day, month**
5		**Where are we- country, county, town, hospital, floor**
	Registration	
3		**Name 3 objects, 1 second to say each, ask patient to recall all 3.**
5		**Serial 7's, (100-7, 93-7 etc.) Stop after 5 correct**
3		**Ask for the aforementioned 3 objects**
	Language	
2		**Name pencil and watch**
1		**Repeat the following 'No ifs, ands, or buts'**
3		**Follow three stage command 'TAKE PAPER IN YOUR HAND, FOLD IT AND PUT IT ON YOUR LAP'**
3		**Read and obey 'CLOSE YOUR EYES'**

Score Results

>25 Normal

>22 Borderline Cognitive Dysfunction

>20 Marked Cognitive Dysfunction

<17 Severe Dementia
